# Individual Data Linkage of Survey Data with Claims Data in Germany—An Overview Based on a Cohort Study

**DOI:** 10.3390/ijerph14121543

**Published:** 2017-12-09

**Authors:** Stefanie March

**Affiliations:** Institute of Social Medicine and Health Economics, Medical Faculty, Otto-von-Guericke-University, 39120 Magdeburg, Germany; stefanie.march@med.ovgu.de; Tel.: +49-391-67-24323

**Keywords:** record linkage, data linkage, Germany, statutory health insurance funds, routine data, primary data, data protection, secondary data, health claims data

## Abstract

Research based on health insurance data has a long tradition in Germany. By contrast, data linkage of survey data with such claims data is a relatively new field of research with high potential. Data linkage opens up new opportunities for analyses in the field of health services research and public health. Germany has comprehensive rules and regulations of data protection that have to be followed. Therefore, a written informed consent is needed for individual data linkage. Additionally, the health system is characterized by heterogeneity of health insurance. The lidA-living at work-study is a cohort study on work, age and health, which linked survey data with claims data of a large number of statutory health insurance data. All health insurance funds were contacted, of whom a written consent was given. This paper will give an overview of individual data linkage of survey data with German claims data on the example of the lidA-study results. The challenges and limitations of data linkage will be presented. Despite heterogeneity, such kind of studies is possible with a negligibly small influence of bias. The experience we gain in lidA will be shown and provide important insights for other studies focusing on data linkage.

## 1. Background

In Germany, customary research practice has had to distinguish between the independent use of primary and secondary data sources [1]. Primary data are data collected and analyzed for a certain scientific purpose, for example, as part of a survey [2]. Such data are collected to answer specific research questions. Among other things, the use of primary data allows researchers to deliberately select a group of interest and record the subjective experience of respondents. In addition to surveys and interviews, qualified staff may also collect standardized medical parameters such as blood pressure measurements. Selection effects may occur if certain groups of test subjects do not participate in primary data collection. Primary data collection requires considerable time, financial and human resources, be it for longitudinal studies, interviewers or postage costs, etc. One should also keep in mind that respondents may exhibit socially desirable response patterns or a recall bias [3,4,5].

Secondary data are data used by researchers beyond their original purpose of collection. In Germany, this includes data from social insurance providers such as statutory health insurers, which are also internationally called health claims data. Such data are generated as part of the administrative everyday business operations of the insurer [2]. Triggered by the scientific use of such data, secondary data analysis has developed in Germany within the past 40-plus years [6,7], with statutory health insurance data being used predominately. Data are available both as case-related, e.g., number of inpatient treatment, and personal-related data, e.g., inpatient treatment per individual, and complete for the group of persons originally concerned because data typically comprise benefits or cost data. Secondary data cover several years and facilitate both retrospective as well as prospective longitudinal analyses. In addition to sector-related analyses, the personal reference contained in the data also facilitates cross-sector studies. However, persons not claiming healthcare services are only included in the denominator, e.g., the number of person at risk including their sociodemographic data is known. Typically, the data also neither includes information about self-paid health services nor information about risk factors or clinical information such as the severity of an illness. By definition no information about people covered by private health insurance is available. Another point of criticism pertains to the validity of detailed information such as outpatient diagnoses [1,4,5,8,9].

Linking different data sources is referred to as data linkage. In computer science, the term “record linkage” is also used [9]. The advantages of different data sources are combined while limitations of individual data sources can be compensated for. These synergy effects create an enriched body of data that forms the basis for answering new research questions [9,10,11,12,13,14,15].

Starting points for a data linkage can be either secondary data or primary data [9,10]. Also, there is no limit to the number of data sources to be linked [9]. Typically, researchers differentiate between linking aggregated secondary data, i.e., a non-person-related linkage with no prior consent given (e.g., [16]), and linking individual person-related data; this paper focuses on the latter [9]. Other differentiations are possible as long as there is at least one commonality. For example, Ohlmeier et al. [17] differentiate between a direct and indirect linkage.

Internationally, linking different data sources is common and accepted practice, especially in the context of population-related issues and the use of secondary data e.g., [18,19,20,21]. For example, Hurd et al. [20] studied the monetary costs of dementia in the United States by linking study data with data of those respondents of the Health and Retirement Study (HRS) who had agreed to the linkage of their claims records. A British study explored predictors of ankylosing spondylitis by interviewing affected patients and linking outpatient and hospital claims data [21]. Internationally, data linkage is also considered a well-established method for external validations e.g., [19,22,23,24,25,26,27,28,29,30,31]. For instance, Hall et al. [23] studied the consistency of self-provided information with respect to the utilization of early screening for prostate cancer and colorectal cancer as documented in the insured person’s records. Another study assessed the consistency of self-reported information with insurance data regarding the prevalence of a number of diseases and found a high rate of agreement regarding information on diabetes mellitus with a Cohen’s Kappa of 0.90 [27]. The authors of the study also acknowledged fundamental differences in prevalence within the data sources depending on the morbidity studied [27]. Koller et al. [29] postulate that neither self-reported information nor information contained in patient records may be used as a gold standard. Consequently, they studied the degree of agreement between both data sources for a number of chronic diseases. They found significant differences and warned against relying on insurance data only when it comes to population-related claims [29]. Carter et al. [28] arrived at similar results. They compared (with prior consent given) self-reported information on any stroke diagnosed by a doctor with personal hospital data and found that prevalence was overestimated in self-reported information. As a consequence, they also call for a “combination of methods (…) to determine prevalence in population-based studies” [28] (p. 2678). To this day, it has not been studied to what extent these findings can be transferred to German circumstances.

Under the condition of encoding personally identifying information, secondary data provided by various German health care providers have been linked without the need for researchers to obtain a person’s written consent since 1999 [32,33]. For example, additional information provided by pathological institutes was integrated into the population-based cancer registry. The method used was a probabilistic linkage, which means probability rates are used as a basis for allocating various sets of data [32]. In contrast, data obtained in a sample by the AOK statutory health insurance fund (Allgemeine Ortskrankenkasse, AOK) of the state of Hesse were linked with data provided by the German Association of Statutory Health Insurance Physicians of the state of Hesse (Kassenärztliche Vereinigung Hessen) for a regional sample survey of insured persons referred to as the regional Statutory Health Insurance Sample of Hesse (Versichertenstichprobe AOK Hessen/KV Hessen). The sample survey used a pseudonymization service as an independent trust agency that pseudonymizes identifying information independent of the research team so that it is possible to carry out a person-related linkage without identifying features [33,34]. Another study on evaluating the progress of femoral neck fractures combined external quality management data with data provided by a statutory health insurer and data collected by the Medical Service of the Health Insurance Funds (Medizinischer Dienst der Krankenversicherung, MDK) [35,36]. The data were also linked and evaluated by an external body [35,36].

Additionally, there are validation studies that link data (called deterministic linkage) if there is an exact match in the data sources [37,38]. Another possibility is a probabilistic linkage [39]. Probabilistic or deterministic linkage will be used especially for databases and registries if several secondary data sources are to be linked. For instance, as part of a feasibility study aimed at evaluating the breast cancer screening program in North Rhine-Westphalia (NRW), data collected by the North Rhine-Westphalia Epidemiological Cancer Registry (EKR NRW) were linked with screening program data and outpatient data provided by the German Association of Statutory Health Insurance Physicians of Westphalia-Lippe (Kassenärztliche Vereinigung Westfalen-Lippe) [40]. Another study linked data collected by EKR NRW with data collected by AOK NordWest (another statutory health insurance fund in Germany) and data pertaining to the disease management program for type 2 diabetes in order to estimate the incidence of cancer in persons with type 2 diabetes [41,42].

A number of German validation studies compare information obtained from different data sources for analyzing of consistency [43,44,45,46]. In addition, there is a substantial body of work focusing on the internal validation of statutory health insurance data [47,48,49]. However, these studies face methodological limits [49,50]. In their introductory article for a special publication on data linkage in Germany, Hoffmann and Abbas [11] (p. 73) came to the conclusion “that one cannot validate secondary data without performing a data linkage”.

Only a very limited number of German studies on health or healthcare provision have linked personal survey data based on the respondents’ informed consent with claims data provided by a single or only a few statutory health insurers. Examples include the Heinz-Nixdorf recall study [51,52], the KORA studies (Cooperative healthcare research in the Augsburg region—KORA); John and Krauth [53] or the Active Health Promotion for Senior Citizens (AGiL) study [15]). Linking data provided by a large number of statutory health insurers creates special challenges. So far relevant experiences have been sparse [9,13,54,55]. On the other hand, linking other social data such as data provided by the Federal German Labor Office (BA) or the German Pension Fund (DRV) has become common practice [56,57,58,59]. Recently, there have been efforts to combine primary data with a host of secondary data sources [9,13,60,61].

Another strategy is to recruit a sample (for instance, with a certain disease) of a statutory health insurance fund and to link these claims data also based on the respondents’ informed consent with survey data. An example of this kind of data linkage is the Linking Patient-Reported Outcomes with CLAIms data for Health Services Research in Rheumatology (PROCLAIR)-study [62,63]. However, data distortions may occur because of lacking consent (consent bias) [64]. Typically, selectivity analyses can be used to quantify such errors [56,57,58,65,66,67].

The linking of survey data with data collected by statutory health insurers (SHI) offers great potential and new opportunities for analyses of today’s and future medical care. A systematic review of the use of secondary data in Germany has generated the following results: ”Data linkage and the improvement of methodological standards could increase the acceptance of claims databased results in policy decision making and early benefit assessment“ [68] (p. 223).

Calls for German data linkage studies in the field of health and medical care research or for external validation purposes have increased steadily [4,10,11,46,50,69,70]. Funding policy also demands the advancement of data linkage methods. The subtitle of the tender for the Medical Information Technology Program of the German Federal Ministry of Education and Research “Linking data—Improving health care” (“Daten vernetzen—Gesundheitsversorgung verbessern”) already spelt it out clearly [71]. Furthermore, the Innovation Committee of the Federal Joint Committee (Gemeinsamer Bundesausschuss (GBA)) explicitly specified “the usage and linkage of routine data to improve healthcare” as a specific research field in its funding announcement [72]. 

In line with this call and based on the example of the lidA study, a German cohort study on work, age, health and work participation, this paper examines individual data linkage as a new method for linking survey data with data collected by a host of statutory health insurers. It also explores the methodological challenges and limitations researchers face in the process. In Germany, the lidA study has played a pioneering role in this respect because, according to the author’s knowledge, for the first time all those statutory health insurers were asked to cooperate with lidA, where at least one policyholder who participated in the study had agreed to the scientific use of health claims data obtained in the baseline survey [54].

## 2. Legal Requirements

Legal prerequisites for data linking depend on the data sources to be linked and the study design, which must demonstrate that there will be direct contact to respondents and obtaining their consent is reasonably feasible.

Statutory health insurance data are considered social data that are subject to special data protection regulations [34]. Pursuant to Article 67 (1) German Social Code (GSC) X [73], personal data is understood as “detailed personal or factual information about an identified or identifiable natural person” that is generated, i.e., collected, processed and used during everyday administrative operations. According to Article 67 (12) GSC X, health-related data are also considered a “special type of personal data” [34]. 

In line with the stipulations of Article 67b GSC X, social data may be used for scientific research under certain conditions. In principle and based on Article 67b GSC X, the person concerned must give their written consent (informed consent) at their free will for the use of their data for research purposes. The consent form must describe the project and the possible consequences of a refusal in writing. Considerable constraints put on researchers that may impede the process of obtaining numerous informed consents as part of the research project must be documented pursuant to Article 67b (3) GSC X. The actual transfer of data for research purposes is permitted for “scientific research in the field of social services or scientific labor market and career research” (Article 75 (1) GSC X) [73].

If researchers can plausibly explain the unreasonableness of obtaining consent and the added value of the project for the public interest, Article 75 GSC X permits exceptions under the condition that the competent federal or state supervisory authority of the institution providing such data permits the project. Applications pursuant to Article 75 GSC X must contain information on who will receive which data from whom and for what purpose and when the data transmitted will be deleted [34]. If a data linkage is planned, researchers must explain in detail what data they intend to link. However, Article 75 GSC X does not stipulate if an application according to said article must be filed with the supervisory authority if the persons concerned have given their informed consent. There are a number of differing practices and interpretations, some of them controversial [34,53,60] which have not been finally settled to this day.

Data owners can transmit social data as absolutely anonymous (“deanonymization is ruled out”), de facto anonymous (deanonymization is only possible with “disproportionate amount of effort”, etc.) or pseudonymized [34] (p. 9). If data are pseudonymized, “certain personal identifiers (e.g., name, address, social security number) are deleted from the data or are replaced by “neutral” non-descriptive study identifiers (…), and “visible” characteristics are aggregated (such as dates of birth DD/MM/YYYY to years of birth YYYY), so that the researchers are given no so-called “unique information” [34] (p. 9). Pursuant to Article 3 of the German Data Protection Act (Bundesdatenschutzgesetz) [74], researchers should always apply the principle of data avoidance and minimization, which means they should only request the social data they actually need to answer their research questions or that are basically new because the data cannot be generated from other data [34].

To comply with data protection aspects when doing a research project using social data, a number of documents pertaining to data protection, such as a data protection concept must be prepared, or agreements between the parties concerned concluded [34]. Moreover, all parties concerned such as in-house and external data protection officers, ethic commissions, supervisory bodies, data owners, research data centers, etc. should be involved in the project at an early stage [34]. It might also be necessary to integrate a trust agency into the data exchange process, be it to store personally identifying data or merge different sets of data [34].

Since 25 May 2016, Regulation (EU) 2016/679 of the European Parliament and the Council of 27 April 2016 on the protection of natural persons with regard to the processing of personal data and on the free movement of such data and repealing Directive 95/46/EC [75] has been in effect. The terms and conditions, binding as of 25 May 2018, are currently being translated into national law [76].

## 3. Individual Data Linkage within the Scope of the LidA Study

The lidA study (www.lida-studie.de) is a sequential cohort study conducted by a consortium made up of the universities of Wuppertal, Ulm and Magdeburg, the infas Institute for Applied Sciences (Institut für angewandte Sozialwissenschaft GmbH), the Institute for Employment Research (IAB) of the Federal Employment Agency (BA) (Institut für Arbeitsmarkt und Berufsforschung (IAB) der Bundesagentur für Arbeit (BA)) as well as the Federal Institute for Occupational Safety and Health (Bundesanstalt für Arbeitsschutz and Arbeitsmedizin (BAuA)) as an associated project partner (lidA consortium). The study explores the relationship between health and aging at work for people born in 1959 and 1965 in Germany. It is funded by the Federal Ministry of Education and Research (BMBF) (funding codes 01ER0806, 01ER0825, 01ER0826, 01ER0827, funding period 1 April 2009–30 December 2015). The ethics commission of the University of Wuppertal has positively reviewed the study. The lidA sample was taken from IAB data available as of the reference date, 31 December 2009. The data are also referred to as integrated employment biographies (IEB data). This explains why the data only pertain to gainfully employed people covered by social insurance. The integrated employment biographies facilitate selectivity analyses with parameters of the population. The sample is representative for the German labor force of the two age groups 1959 and 1965. During a computer-assisted interview (CAPI), participants were asked about work, age, labor participation, and health in two rounds of interviews in 2011 and 2014. CAPI data comprise, among others, information on current employment, work exposure, subjective psychological and physical health and socio-demography [60,77,78,79]. A description of the baseline sample is included in Hasselhorn et al. [78].

In addition to CAPI data, the study individually linked IEB as well as statutory health insurance data provided by a number of different statutory health insurers with survey data if consent had been obtained. In the case of statutory health insurance data, the data were also provided aggregated in the form of a work-health matrix with the key variables age, sex, and work characteristics [60]. IEB data have retrospectively been available for the old federal states since 1975 and for the new federal states since 1991. The data include job histories of people during times they paid social insurance contributions as well as information on periods without gainful employment (e.g., periods of parental leave) or periods during which said person received benefits from the Federal Employment Agency [60]. In addition to master data, statutory health insurance data comprise information regarding sick leave, outpatient and inpatient data (e.g., ICD coded), data on outpatient drug prescription and treatments/remedies received [60,78]. Except for insurer-specific exceptions, the data are available for 2008 through 2013.

### 3.1. Data Protection Procedures

From a data protection perspective, the data used in the lidA study are considered social data in line with Article 75 GSC X. Therefore, applications according to said article had to be filed with the competent supervisory authorities. The Federal Ministry for Labor and Social Affairs (BMAS) is responsible for approving the use of IEB data, both with respect to taking the sample as well as individual IEB data to be linked. In comparison, there are a number of supervisory authorities on the federal and state level overseeing the use of statutory health insurance data (SHI data). Contrary to IEB data, the health insurer concerned must file an application according to Article 75 GSC X with the relevant supervisory authority. To this end, health insurers are provided with a sample application in order to guarantee uniformity in lidA. In the above case, the Institute of Social Medicine and Health Economics (ISMHE), as a research body, filed an application with the supervisory authority on the federal level on behalf of the health insurers. All statutory health insurers filing an application in their capacity as data providers were expected to refer to the mentioned application in their own application, which was still required pursuant to Article 75 GSC X. However, this application had to be identical to the one the ISMHE filed [60]. This procedure shortens the processing time of individual applications. Applications cover the use of both individual data as well as aggregated statutory health insurance data [34,60].

To perform an individual data linkage (CAPI–IEB, CAPI–SHI) and remain in the cohort (willingness to participate in the follow-up), respondents had to give their informed written consent for which they were asked during the interview [77]. Prior to the survey, the lidA consortium thoroughly discussed how to phrase information and design consent forms for participants and the best time to asked during the CAPI to get the best possible response [60]. “Under such circumstances, researchers, together with data protection officers, must try to strike a balance in their wording of information for participants and the informed consent that is easily understandable and concise on the one hand and on the other hand complies with data protection regulations as it provides sufficient and clear information about the intended use of the data.” [9] (p. 185).

All institutions of the lidA consortium have a data protection concept in place that regulates the use of personal data. In addition, the consortium concluded an additional data protection agreement with the IAB to stipulate procedures and the transfer of data to consortium partners within the lidA study. Another additional data protection agreement governing the use of health insurance data was concluded within the consortium [60]. Prior to the study, all relevant stakeholders, for example external data protection officers of the health insurers concerned or the competent supervisory bodies were involved in the drafting process of all data protection documents and in coordinating procedures and drafting contracts with data providers [34,60].

Based on a complex process using several different study identification numbers, a trust agency guaranteed that the researchers of the lidA consortium work with pseudonymized data only [60,77].

### 3.2. Statutory Health Insurers

About 87 percent of all Germans are covered by statutory health insurance while 11 percent have private health insurance. The remaining population receives free healthcare, for example soldiers [80]. In 1996, the Health Care Structure Act of 1993 introduced the free choice of health insurer for people having statutory health insurance with only a few exceptions for e.g., special groups like the agricultural profession. Until then, people covered by statutory health insurance were allocated to an insurer depending on their profession as well as on regional factors [81]. The Act to Strengthen Competition in Statutory Health Insurance permits the merger of different health insurers which some health insurers have taken advantage of since 2009 [82].

Since 1 January 2009, a uniform premium has been in effect for statutory health insurers with health insurers having the option of charging extra premiums. This has led to a migration of insured persons to other insurers as some have experienced [82]. In a survey conducted by the Scientific Institute of the AOK (WIdO) in 2011, some 22 percent of respondents indicated that they had changed their health insurer because of an extra premium charged. Especially young and healthy people tend to change health insurers [83]. The change of health insurance has an additional effect on conducting studies that intend to link individual health insurance data. In case of a health insurance change, data can only be retrieved until the end of the membership period with the health insurer concerned. The data generated after the change of health insurer will be excluded from use if no new consent has been obtained because any informed consent given only applies to the person’s health insurer at the time of the CAPI. That is why researchers asked for renewed consent to link data during the second wave of the lidA survey if a respondent indicated during the interview that s/he changed health insurers [79]. What makes the process even more difficult is the fact that the electronic health card with an insurer-independent new personal life-long (unique) insurance number was introduced on 1 January 2011 with a transition period until 31 December 2014 [84]. 

Research using statutory health insurance data provided by individual health insurers may lead to possible selection effects [54] due to differences in the policyholder base and morbidity [85,86,87]. A systematic review of the literature also shows clear differences between people having statutory or private health insurance [88]. Relating the results of individual health insurers to the overall population has been discussed critically in recent years [4,87]. That is the reason why the lidA study contacted all health insurers for whom at least one informed consent had been obtained during the first round of interviews [54].

The number of statutory health insurers has drastically fallen from 1815 to 112 since the 1970s (as of 30 November 2017) [89,90]. This means that since the start of the lidA study in 2009, the number of health insurers has decreased by 86.

In the long run, mergers will lead to a lower number of health insurers to be contacted and therefore to fewer contacts which will also cut acquisition time. This in return will create more homogeneous data sets [54]. The current total number of 13 supervisory authorities to be contacted (as of 24 October 2017; March et al. [54] still counted 15) is closely related to the merger of health insurers because depending on the sphere of competence of a health insurer for research involving secondary data, different supervisory authorities on the federal and state level have competence. Competent supervisory authorities must also grant permission for mergers of health insurers, which not only ties up time, organizational and human resources of the health insurer concerned but also of the supervisory authority responsible for processing applications according to Article 75 GSC X [54]. Within lidA, informed consents given can be allocated to a total of 95 health insurers (as of 2014). Following a large acquisition effort, contracts with eleven statutory health insurers were signed [91] and therefore eleven applications according to Article 75 GSC X were filed with eight different supervisory authorities. Despite the use of a standard form, variations occurred in individual approval so that the most restrictive approval formed the basis for the further use of statutory health insurance data [54]. In the further course of the lidA project, the ISMHE or the health insurer concerned contacted some supervisory authorities again in an effort to streamline consent afterwards, for example, with regard to deletion deadlines. This is a tedious process but well worth the effort with regard to other permissions and it was successful in lidA.

Recently, calls for implementing cross-insurance data sets have become louder [43,54,68,87]. In Germany the implementation of a research data center analogous to that of other social security providers such as the BA or DRV would actually make the access to statutory health insurance data easier. What is more, health insurance changes by policyholders could be accounted for [54].

### 3.3. Validity

**Figure 1:**
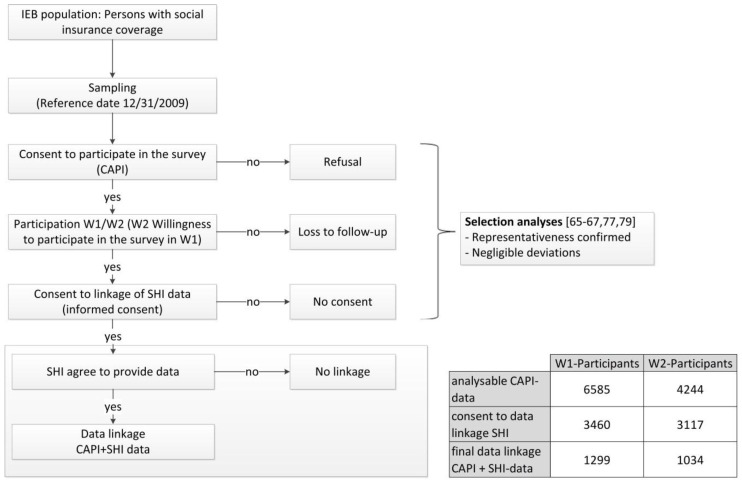
Potentially selection effects within the lidA study [55,77].

A score of different selection effects may occur in longitudinal studies with an intended data linkage. As part of the lidA study the following effects could occur:

(1) Consent by respondents to participate in the study (participation vs. refusal),
(2) Consent by respondents to participate again (willingness to participate in the follow-up),
(3) Renewed participation in the survey (participation vs. loss to follow-up),
(4) Consent by respondents to have their secondary data linked (participants consent vs. no consent),
(5) Willingness of health insurers concerned to provide data (provision of health insurance data vs. no provision).” [55] (p. 105) (see Figure 1).

In total, the baseline sample comprised 6585 respondents. This equals a response rate of 27.3 percent according to the standards of the American Association for Public Opinion Research (AAPOR) [77]. In the sample the marginal distributions of the participants’ socio-demographic characteristics matched those of the total population (point 1). Deviations found are negligible confirming the representativeness of the study [77] (see Figure 1).

Almost 85 percent declared their willingness to continue participating in the study, approx. 75 percent agreed to the linkage of IEB data, and some 55 percent gave their consent to have statutory health insurance data linked [77]. In 2014, 4244 interviews were conducted during the second survey wave. Consent to link health insurance data rose to 63 percent [79]. In other German studies, consent rates for data linkage differ significantly. The AGIL study reached a consent rate of 100 percent because participants gave their consent by registering for the integrated healthcare project [15]. The SHIP study (Study of Health in Pomerania) achieved a consent rate of 94 to 98 percent for the intended linkage of secondary data because participants were asked in one of the follow-ups after a basis of trust had already been developed and/or directly at the study center [61]. The pre-test of the German National Cohort had a similar outcome [92]. However, when SHIP requested consent at a later stage by mail, only about 60 percent of respondents agreed [61]. In a mailed request with a written reminder and subsequent phone call, John and Krauth [53] achieved a consent rate of nearly 78 percent. Again, participants had already actively taken part in the first study [53]. Scholten et al. [93] had a response rate of about 50 percent for a survey conducted in North Rhine-Westphalia among breast cancer patients. The consent rate did vary though with respondents covered by AOK Rheinland/Hamburg (another statutory health insurance fund in Germany) giving their consent at a rate of about 90 percent compared to 76 percent for the remaining respondents [93]. Cruise et al. [94] report consent rates of 71 percent for linking survey data with medical records, with consent given during the interviews for a longitudinal study carried out in Great Britain. In another study from Great Britain, the consent rate reached just 41 percent remaining well below that of lidA although similar to the lidA study consent was requested during CAPI interviews [95].

As part of the lidA study, consent behavior was examined for possible influencing factors. The analyses found only marginal selection effects [65,66,67,77,79] (see Figure 1).

If consent was granted, IEB data could be completely linked with CAPI data, which means without any mismatch because the sample was taken from the same data source [60]. Also, there was only one data owner involved [54]. This, however, does not apply to the linking of health insurance data. Because consent is spread over a large number of health insurers it does not suffice to just compare persons willing to give their consent with those not willing to do so (point 4). That is why a final analysis examined the performed linkage of health insurance data (linkage probability, point 5) by way of a selectivity analysis for each survey wave using multivariate binary logistical regression analyses and prevalence comparisons (with vs. without health insurance data linkage) (see Figure 1). The question to be answered was if eleven health insurers present a sufficient number of contractual partners to guarantee that the linkage of health insurance data with CAPI data did not result in any distortions. To this end, questions regarding socio-demographic characteristics, work and health as well as CAPI information comparable to data provided by statutory health insurers such as information on work inability or a change of health insurer were integrated as independent variables in a model to explain the data linkage reached. Despite signed contracts, only ten health insurers provided data [55].

In 21 percent of the cases (*n* = 1299), health insurance data was linked to respondents of the first survey wave (W1) while that number rose to 24 percent (*n* = 1034) for the second survey wave (W2) [55,96]. In the end, in both models only the qualification to attend a technical university compared to having obtained a secondary school leaving certificate (W1 OR: 1.63 (95% CI 1.30; 2.05); W2 OR: 1.83 (95% CI 1.41; 2.38)) and the two characteristics complexes “profession” based on the Blossfeld classification [97] and “education” had a significant impact while the validity of the model (R2 according to Nagelkerke) is low at 0.044 for W1 and 0.047 for W2. However, a low model validity expressed by a low R squared value is desirable for this selectivity analysis. In principle, selectivity effects are negligible in this analysis (except for education and profession) although only data provided by ten health insurers were used in the analysis. The authors are not aware of any studies where such a selectivity analysis for the linkage of health insurance data has already been performed [55].

## 4. Discussion

As part of the lidA study, analyses are currently performed examining the cross validity of certain information included in lidA survey data and health insurance data, such as information on certain diseases or risk factors (hypertonia, smoking, obesity/adiposity, etc.) [98,99,100]. Initial analyses show that health insurance data reflect health risks such as smoking or the use of opportunities for health improvement only to a very limited extent [98]. On the other hand, it is easy to present adiposity accurately [99]. A comparison of information on the prevalence of hypertonia—self-reported prevalence in CAPI W1 and W2 versus administrative prevalence as a combination of information of individual sectors of health insurance data—shows medium consistency with a Cohen’s Kappa of 0.56 [101]. This is an indication that information obtained from both data sources is necessary and the data linkage beneficial.

The lidA study demonstrates that it is possible to individually link a score of secondary data without distortions, especially with regard to health insurance data provided by several statutory health insurers. A comparison with other German studies [15,53,61,93] demonstrates that the way and point in time that consent is obtained may influence the consent rate.

However, getting a large number of statutory health insurers involved and complying with data protection requirements is very time-consuming and requires substantial resources from the very start which must be figured into study applications and designs. Also, changes of health insurers by respondents cannot be accounted for. This fact should also be remembered during the planning stage of a study. In the lidA study, the share of respondents indicating a health insurance change within the past year was between four to six percent [55]. A similar figure of 4.2 percent was recorded during the pre-test of the German National Cohort [92]. Researchers should also get in contact with all relevant actors at an early stage [9,60]. Late contact may result in additional requirements that may void informed consents already obtained [93]. Using the right study design will also help avoid or assess numerous possible selection effects. Selectivity analyses should therefore be standard practice for studies involving data linkage [55].

For the time being, in Germany the heterogeneity of health insurers will persist, making it necessary to cooperate with a large number of health insurers. Consequently, cross-insurance data sets—to be obtained from a research data center yet to be founded—would present a desirable alternative [54,55,60].

## 5. Conclusions

The lidA study and especially its methodology of data linkage are on a par with the state-of-the-art internationally. In 2014, Ferrie wrote ([102], p. 1690): “The existence of lidA (…) brings German epidemiological research into line with that in other countries, which for many years have enjoyed the advantages of linked individual survey data and register based data”.

The lidA study provides important experience with regard to the individual linkage of survey and secondary data that other studies can build on. The German National Cohort has recruited some 200,000 participants [103] who have been asked to give their consent to have a range of secondary and registry data linked, including statutory health insurance and IEB data as well as private health insurance data and data collected by DRV [12,13].

In Germany, data linkage has become a method of choice for secondary data analysis as a glimpse into standard German literature reveals, e.g., [2,104,105].

In its opinion on the scientific and social importance of population-wide longitudinal studies (“Wissenschaftliche und gesellschaftspolitische Bedeutung bevölkerungsweiter Längsschnittstudien”) published in May 2016, the Academy of Sciences Leopoldina together with acatech, the German Academy of Technical Sciences, and the Union of German Academies of Sciences [106] called for awareness of the “untapped potential of data linkage” ([106], p. 61). Their recommendation states “4.4 Legal and technical opportunities for linking survey and administrative data need to be expanded (“data matching and linkage”) (…) as well as the safeguarding of data protection and participants ethics. All this requires additional resources that need to be figured into study funding.” ([106], (pp. 73–74). 

To avoid a black box of record linkage [107] in Germany, a team of experienced scientists led by the author is currently working out a status quo of data linkage in Germany. In addition to individual methods and types of data linkage and legal aspects, the team also focuses on software tools, practical examples, and quality assurance tips [108]. This is only a first step to gain a comprehensive understanding of the situation in Germany.
